# Pediatric Methemoglobinemia Following Accidental Ingestion of Sodium Nitrite Used as a Sausage Preservative: A Case Report

**DOI:** 10.1002/ccr3.71647

**Published:** 2025-12-10

**Authors:** Sharareh Babaie, Azin Momeni

**Affiliations:** ^1^ Department of Pediatric Intensive Care Unit, Imam Hossein Children's Hospital Isfahan University of Medical Sciences Isfahan Iran; ^2^ Department of Pediatrics, Child Growth and Development Research Center, Research Institute for Primordial Prevention of Non‐Communicable Disease Isfahan University of Medical Sciences Isfahan Iran

**Keywords:** methemoglobinemia, methylene blue, pediatrics, poisoning, sodium nitrite

## Abstract

Accidental sodium nitrite ingestion can cause life‐threatening pediatric methemoglobinemia unresponsive to oxygen therapy. Early recognition and prompt methylene blue treatment are lifesaving. Public awareness about the hazards and safe amounts of sodium nitrite in homemade sausage is essential to prevent poisoning in children.

## 1 Introduction

Methemoglobin is a form of hemoglobin that has been oxidized, changing its heme iron configuration from the ferrous (Fe2+) to the ferric (Fe3+) state [1]. Unlike normal hemoglobin, methemoglobin is unable to efficiently transport and release oxygen to the tissues. This causes a leftward shift in the oxygen–hemoglobin dissociation curve and decreased oxygen delivery to the body [2].

Methemoglobinemia can be congenital or acquired. Most cases of methemoglobinemia are acquired, resulting from increased methemoglobin formation induced by various exogenous substances. Sodium nitrite is one of these substances, used as a food preservative. Poisoning with this preservative has been reported in a few cases of suicides in adolescents [3, 4, 5], but unintentional poisoning in children is rare [6]. Here, we present a case of methemoglobinemia induced by unintentional poisoning with homemade sausage preservative (sodium nitrite) in a 4‐year‐old girl.

## 2 Case Presentation

### 2.1 Case History/Examination

A previously healthy 4‐year‐old girl presented with a 2‐h history of progressive cyanosis following accidental ingestion of sodium nitrite. Approximately half a teaspoon of the substance, used by her mother as a preservative in homemade sausage, had been dissolved in a glass of water, which the child drank entirely due to inadequate safety precautions.

One hour after ingestion, she vomited food contents without blood and developed cyanosis involving her lips and nail beds. Her dyspnea worsened. This prompted her family to bring her to our hospital 2 h after ingestion.

Upon arrival at the emergency department, initial vital signs showed hypoxemia with an oxygen saturation detected by pulse oximeter (SpO_2_) of 85%. Her blood pressure was 95/50 mmHg, and she was afebrile with an oral temperature of 36.7°C. She exhibited tachypnea (respiratory rate: 33 breaths/min) without the use of accessory muscles. Her heart rate was 130 beats/min, attributed to agitation and respiratory distress.

**FIGURE 1:**
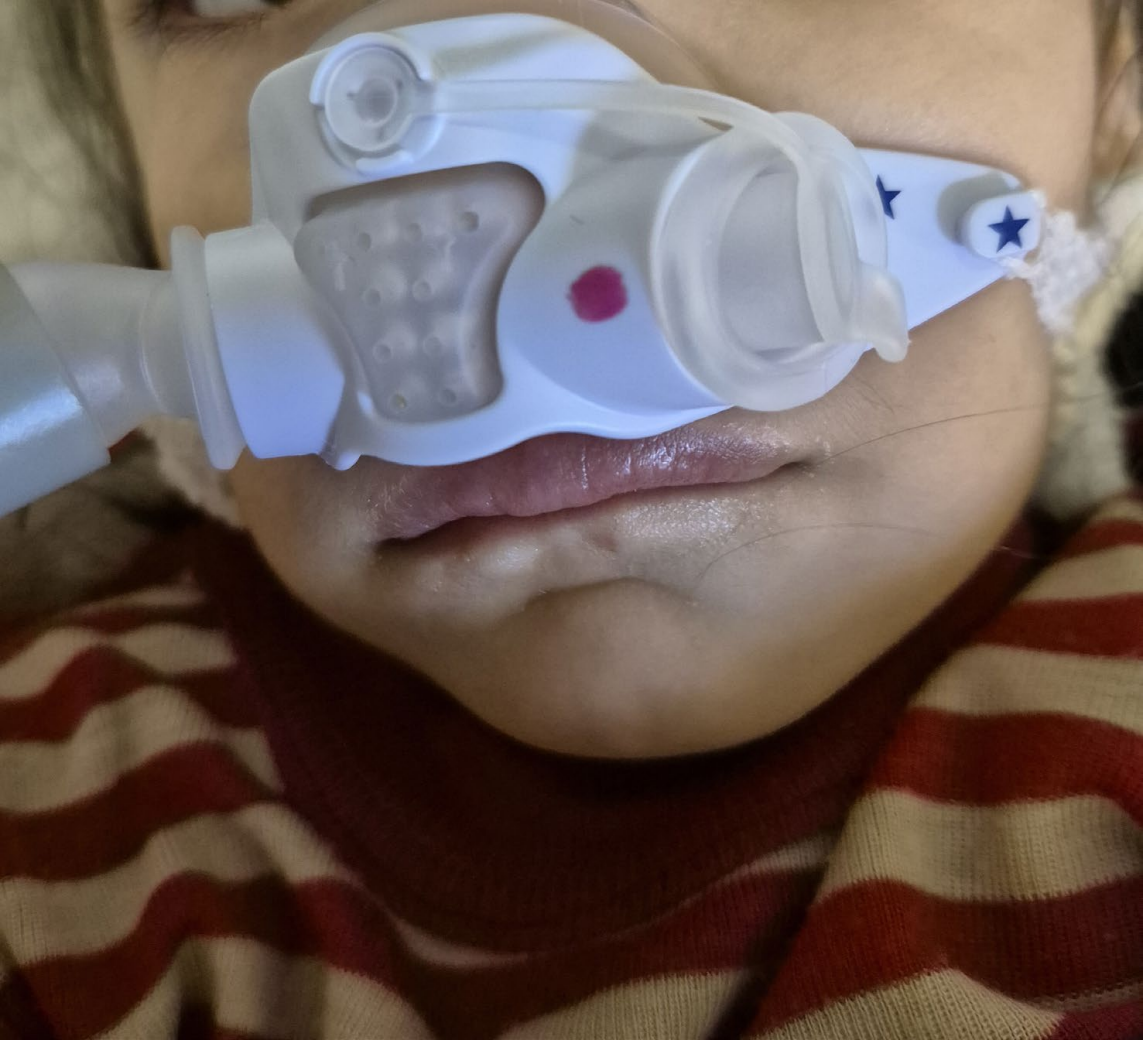
Bluish‐gray cyanosis of the lips and perioral region in a 4‐year‐old girl following acute sodium nitrite ingestion.

On physical examination, the patient was alert and agitated, with cyanosis of the skin, lips, and nail beds, manifesting a grayish‐blue discoloration, but did not appear toxic (Figure 1). Breath sounds were clear and equal bilaterally. There was no organomegaly, and peripheral perfusion was normal. The remainder of the physical examination was unremarkable.

### 2.2 Methods (Diagnosis, Investigations, and Treatment)

Laboratory and radiographic investigations revealed a normal complete blood count: white blood cell count of 11.7 × 10^9^/L (with 40% neutrophils), hemoglobin concentration of 111 g/L, and platelet count of 327 × 10^9^/L. Serum electrolytes, renal and liver function tests, glucose‐6‐phosphate dehydrogenase (G6PD) level, and reticulocyte count were all within normal limits. Arterial blood gas (ABG) analysis could not be obtained due to the child's uncooperativeness; therefore, a venous blood gas (VBG) analysis was performed. It revealed a pH of 7.49, decreased partial pressure of carbon dioxide (PvCO_2_ 24 mmHg), reduced bicarbonate (17.7 mEq/L), negative base excess (−5.6 mmol/L), normal partial pressure of oxygen (PvO_2_ 38 mmHg), and high oxygen saturation (SvO_2_ 82%). Based on the VBG, acute respiratory alkalosis was the major disorder, but because HCO₃^−^ was lower than the expected compensatory response, mild metabolic acidosis also existed. Lactate level was 3.2 mmol/L. Methemoglobin level testing was not available at our hospital. The chest X‐ray was normal.

Oxygen therapy via a non‐rebreather mask was initiated but failed to improve her oxygenation. One hour later, her SpO_2_ dropped to 60%, prompting initiation of continuous positive airway pressure (CPAP) and transfer to the pediatric intensive care unit (PICU). Despite CPAP, her oxygen saturation remained around 85%, indicating minimal response to supplemental oxygen.

### 2.3 Conclusions and Results (Outcome and Follow‐Up)

The patient was initially treated with intravenous vitamin C at a dose of 15 mg/kg because methylene blue was not available at our hospital. Four hours after admission, methylene blue became available and was administered intravenously at 1.5 mg/kg over 10 min. Twenty minutes after the methylene blue infusion, her oxygen saturation began to increase. CPAP support was discontinued approximately one hour later, and within two hours, she was stable without the need for supplemental oxygen.

**FIGURE 2:**
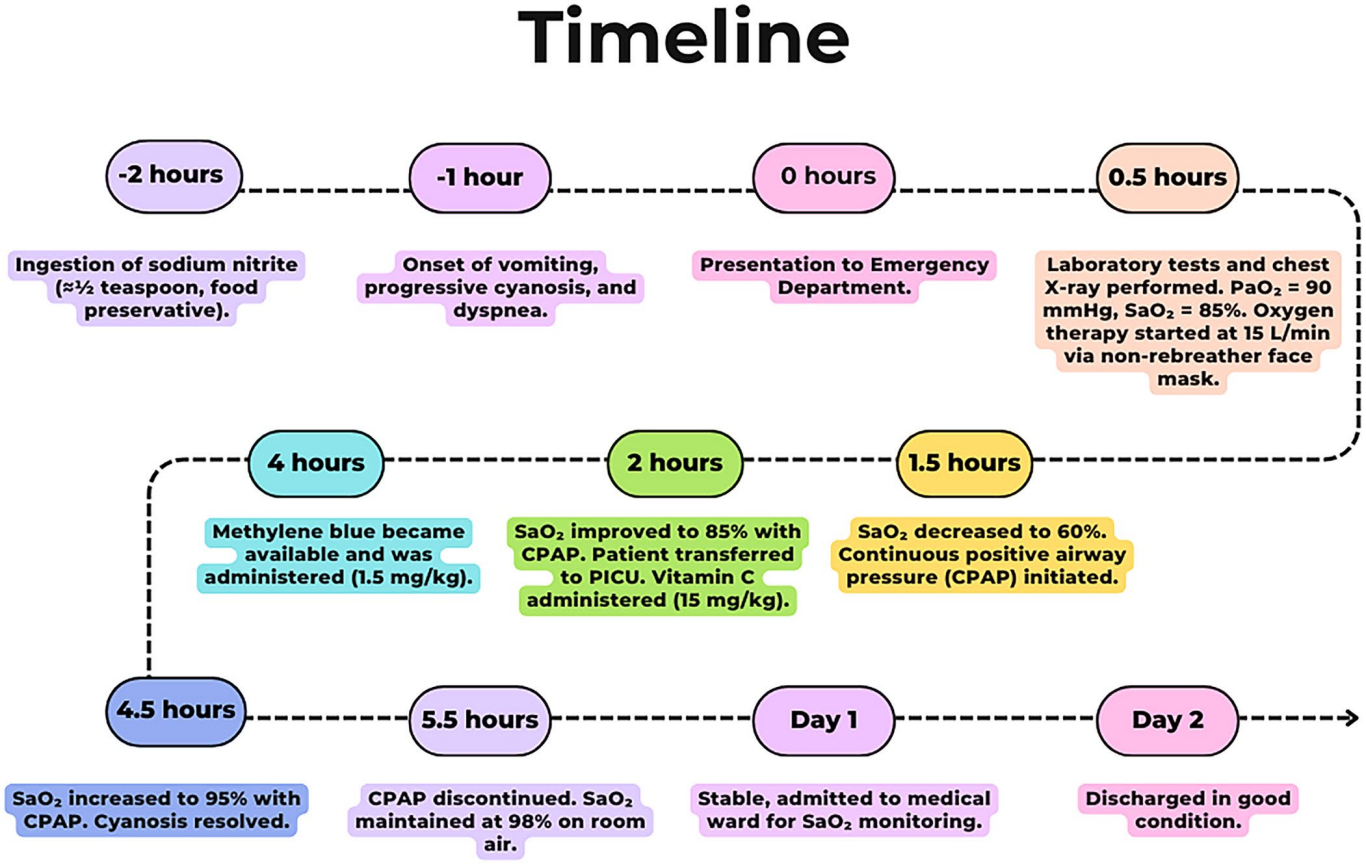
Timeline of clinical presentation, diagnostic findings, treatments, and recovery in the same patient with acute sodium nitrite poisoning.

The patient was monitored and remained stable throughout the following day. She was discharged in good condition 48 h after admission (Figure 2).

## 3 Discussion

Sodium nitrite is used as a food preservative, and cases of intentional ingestion have resulted in severe methemoglobinemia [3, 4, 5]. Sodium nitrite is also used for treating cyanide poisoning and therapeutically inducing the formation of methemoglobin, which preferentially binds cyanide [7]. So far, accidental sodium nitrite poisoning has been reported in only seven cases in children under 10 years of age [2, 6], and this report presents the first documented case from Iran involving a previously healthy 4‐year‐old child. She developed progressive cyanosis and respiratory distress 2 h after accidentally ingesting sodium nitrite, which was used as a preservative in homemade sausage. Despite oxygen therapy and CPAP, her oxygen saturation remained at 85%. Confirmatory testing for methemoglobinemia was unavailable; therefore, based on the history and clinical findings, initial treatment with intravenous vitamin C was administered, followed by methylene blue once it became available. This led to rapid clinical improvement. She stabilized without the need for further respiratory support and was discharged in good condition 48 h later.

Methemoglobinemia should be suspected in patients with unexplained cyanosis or hypoxia that does not improve with supplemental oxygen, particularly following exposure to oxidizing agents. Key diagnostic clues include a persistent oxygen saturation (SpO_2_) around 85%, normal arterial oxygen pressure (PaO_2_), and the presence of pale, gray, or blue skin, lips, and nail beds. Blood may appear dark red, chocolate, or brownish‐blue and fail to return to normal color with oxygenation. Prompt recognition is critical, as methemoglobinemia is rare but potentially life‐threatening, and symptom severity often correlates with methemoglobin level [8, 9, 10]. In this case, the patient presented with cyanosis of the skin, lips, and nail beds, manifesting as grayish‐blue discoloration following the ingestion of sodium nitrite, which did not improve with supplemental oxygen and was associated with a persistent SpO_2_ of approximately 85%. We obtained a VBG instead of an ABG. Although there is correlation between VBG and ABG in measurement of pH, PCO₂, and HCO₃, there are no established venous‐to‐arterial conversions for SO₂ or PO₂. However, in the patient's VBG, SvO₂ was elevated and PvO₂ was within the normal range. This presents a paradox, as the patient was clinically cyanotic and hypoxic, yet her VBG appeared consistent with that of a well‐oxygenated individual. A possible explanation is that in methemoglobinemia, hemoglobin returns to the venous circulation still largely saturated with oxygen due to impaired unloading [2]. As a result, oxygen remains dissolved in the plasma of venous blood. While we had no difficulty diagnosing our patient due to the clear history of sodium nitrite ingestion, normal or high PvO₂ and SvO₂ in a cyanotic patient should be considered a red flag.

Methylene blue (MB) is the first‐line treatment for symptomatic acute toxic methemoglobinemia, especially when methemoglobin levels exceed 30%, due to its rapid and well‐established effectiveness. It may also be used for symptomatic patients with levels between 20% and 30%, particularly if they have underlying cardiopulmonary conditions [11]. The standard dose is 1–2 mg/kg IV over five minutes, with a possible second dose after one hour if levels remain elevated; however, total doses above 7 mg/kg are generally avoided due to the risk of hemolysis [12]. MB is contraindicated in individuals with G6PD deficiency or those on serotonergic medications, in which case ascorbic acid (vitamin C) is used as an alternative [13, 14]. Ascorbic acid can also be used when MB is unavailable, though its effect is slower, with improvement typically seen over 1–3 days [15]. In resource‐limited settings where methemoglobin testing is not available, treatment decisions are often based solely on clinical signs and symptoms, prompting empirical use of MB and/or vitamin C. In this case, due to the unavailability of methemoglobin level testing, treatment was initiated based solely on the patient's clinical presentation. Vitamin C was administered first because methylene blue (MB) was not available at our hospital and had to be obtained from the regional poisoning center. Once MB became available, it was added to the treatment regimen, and the response was rapid and favorable.

As the use of homemade sausage is increasing in Iran and its production is becoming a cottage industry, this case underscores the importance of educating people about the potential risks of sodium nitrite as a preservative and keeping it away from children to prevent poisoning. In addition, people should be made aware of the safe concentrations of this preservative when preparing sausages. Clinicians should consider acquired methemoglobinemia in patients who exhibit unexplained cyanosis or hypoxia unresponsive to supplemental oxygen. Given the potentially life‐threatening nature of this condition, it is essential that MB be readily available in all healthcare facilities. Timely administration of MB is critical for optimizing patient outcomes in cases of methemoglobinemia due to poisoning.

## Author Contributions

**Sharareh Babaie:** conceptualization, data curation, resources. **Azin Momeni:** supervision, writing – original draft, writing – review and editing.

## Funding

The authors have nothing to report.

## Ethics Statement

A comprehensive verbal description of the objectives of the study was given to the patient, and written informed consent was obtained from the parents.

## Consent

Written informed consent for the publication of clinical details and accompanying images was obtained from the patient's parents.

## Conflicts of Interest

The authors declare no conflicts of interest.

## Data Availability

The datasets generated and/or analyzed during the current study are available from the corresponding author on reasonable request.
